# Anti-cancer effect of Annona Muricata Linn Leaves Crude Extract (AMCE) on breast cancer cell line

**DOI:** 10.1186/s12906-016-1290-y

**Published:** 2016-08-24

**Authors:** Syed Umar Faruq Syed Najmuddin, Muhammad Firdaus Romli, Muhajir Hamid, Noorjahan Banu Alitheen, Nik Mohd Afizan Nik Abd Rahman

**Affiliations:** Faculty of Biotechnology and Biomolecular Sciences, Universiti Putra Malaysia, Serdang, Selangor Malaysia

**Keywords:** *Annona muricata* Linn, Breast cancer cell line, Potency, Leaf aqueous extract, Apoptosis, Anti-metastatic, Immune systems, Inflammation

## Abstract

**Background:**

*Annona muricata* Linn which comes from Annonaceae family possesses many therapeutic benefits as reported in previous studies and to no surprise, it has been used in many cultures to treat various ailments including headaches, insomnia, and rheumatism to even treating cancer. However, *Annona muricata* Linn obtained from different cultivation area does not necessarily offer the same therapeutic effects towards breast cancer (in regards to its bioactive compound production). In this study, anti-proliferative and anti-cancer effects of *Annona muricata* crude extract (AMCE) on breast cancer cell lines were evaluated.

**Methods:**

A screening of nineteen samples of *Annona muricata* from different location was determined by MTT assay on breast cancer cell lines (MCF-7, MDA-MB-231, and 4 T1) which revealed a varied potency (IC_50_) amongst them. Then, based on the IC_50_ profile from the anti-proliferative assay, further downward assays such as cell cycle analysis, Annexin V/FITC, AO/PI, migration, invasion, and wound healing assay were performed only with the most potent leaf aqueous extract (B1 AMCE) on 4 T1 breast cancer cell line to investigate its anti-cancer effect. Then, the in vivo anti-cancer study was conducted where mice were fed with extract after inducing the tumor. At the end of the experiment, histopathology of tumor section, tumor nitric oxide level, tumor malondialdehyde level, clonogenic assay, T cell immunophenotyping, and proteome profiler analysis were performed.

**Results:**

*Annona muricata* crude extract samples exhibited different level of cytotoxicity toward breast cancer cell lines. The selected B1 AMCE reduced the tumor’s size and weight, showed anti-metastatic features, and induced apoptosis in vitro and in vivo of the 4 T1 cells. Furthermore, it decreased the level of nitric oxide and malondialdehyde in tumor while also increased the level of white blood cell, T-cell, and natural killer cell population.

**Conclusion:**

The results suggest that, B1 AMCE is a promising candidate for cancer treatment especially in breast cancer and deserves further research as an alternative to conventional drugs while also stressed out the selection of soursop sample which plays a significant role in determining its potential therapeutic effect on cancer.

**Electronic supplementary material:**

The online version of this article (doi:10.1186/s12906-016-1290-y) contains supplementary material, which is available to authorized users.

## Background

Breast cancer is one of the leading cancer affecting women as over 1 million women worldwide are diagnosed with this disease each year [1]. Despite the current drugs present that manage to suppress the tumor growth, there is an urgent need to explore alternative strategies to overcome several limitations in treating breast cancer including the metastasis of cancerous cells which is the leading cause of mortality and morbidity, increasing the sensitivity of immune system response, and reducing the inflammation caused by cancer. With the advance of research to date, many medicinal plants have been subjected to scientific scrutiny where their secondary metabolites/bioactive compounds are discovered to have the anticancer effect potential. *Annona muricata* Linn which belongs to the Annonaceae family has been used in traditional medicine to treat various ailments including fever, rheumatism, cancer, and also as sedative, insecticide, and immunosuppressive activity [2]. Intensive research on the chemical composition of the leaves [3] and seeds [4] lead to the finding of acetogenin compounds which explains the therapeutic effects it possessed. Acetogenin (ACG) is characterized by its unbranched C_32_ or C_34_ fatty acid with a γ-lactone at the end of the cytoskeleton [5]. This molecular structure is a very potent compound against cancer as it deprives the highly energy demanding cancer cells from adenosine triphosphate (ATP) supply via the disruption of mitochondrial electron transport system, hence resulting in apoptosis [6, 7]. The production of secondary metabolites is actually a response by plants to cope with the harsh or ever changing environments. It has been reported that plant of similar species collected from different locations has a varied level of secondary metabolites among them [8] which indicates that the production of the bioactive compounds in the soursop plant could also vary thus, affecting its potency against cancer cell. As that notion has not yet been tested, therefore, the purpose of this study was to screen the cytotoxicity level of the *Annona muricata* crude extract (AMCE) against the breast cancer cell lines (MCF-7, MDA-MB-231, and 4 T1) as well as to further evaluate the anticancer effect possessed by the selected (most potent) AMCE on 4 T1 cancer in vitro and in vivo.

## Methods

### Preparation of *Annona muricata* Crude Extract (AMCE)

Samples of *Annona muricata* leaves were obtained from the *Annona muricata* cultivars in Johor, Melaka, Negeri Sembilan, Selangor, Perak, and Perlis in the months of September to November 2014. The plant was identified and deposited with a voucher number by Science Officer Lim Chung Lu from the Forestry Division, Forest Research Institute Malaysia. Details of the sampling sites and voucher number of each sample are shown in Additional file 1: Table S1. All of the 19 samples of old mature *Annona muricata* leaves were air-dried for a week before being ground to a powder using a grind mill. Later, about 10 g of each powdered samples were transferred into a Schott bottle containing 200 mL of cold sterile distilled water. The samples were incubated for 3 days with frequent agitation using an orbital shaker at room temperature. The mixture was then, filtered to discard any solid material/marc. Finally, the filtrate extract was dried using the freeze dryer/ lyophilizer machine to give the end product (AMCE).

### Cell culture

The cell lines, MCF-7, MDA-MB-231, 4 T1 and MCF-10A were obtained from the American Type Culture Collection (ATCC, Manassas, VA, USA). The MCF-7 and 4 T1 cells were maintained in RPMI 1640 medium while MDA-MB-231 cell was maintained in DMEM medium. Both media were supplemented with 10 % Fetal Bovine Serum (FBS) and 1 % Penicillin/Streptomycin. MCF-10A on the other hand, was maintained in DMEM-F12 medium supplemented with hydrocortisone (0.5 μg/mL), insulin (10 μg/mL), hEGF (20 ng/mL) and 10 % FBS. The cells were grown in a humidified incubator at 37 °C in the presence of 5 % CO_2_. The cell was passaged upon reaching 70 % confluency.

### MTT assay

The proliferation of the cells or cell viability was assessed by the 3-[4,5-dimethylthiazol-2-yl]-2,5 diphenyltetrazolium bromide (MTT) dye reduction as described by Zhi-Dong et al [9]. The cytotoxic potential of the crude extract samples could be determined from this assay based on the IC_50_ generated. A hundred microliter of cells at a concentration of 0.8 × 10^5^ cells/well were placed into a 96-well plate and maintained in the respective medium (RPMI/DMEM) for 24 h. The following day, *Annona muricata* crude extract (AMCE) was added to the wells and then, incubated for 72 h. MTT solutions (5 mg/ml) was added at a volume of 20 μL into each wells and incubated for 3 h. Later, the solutions were removed from wells and 100 μL of DMSO were added to solubilize the formazan crystals. Finally, the plate was read using an ELISA plate reader at a wavelength of 570 nm (Bio-tek Instruments, USA).

### Annexin V/FITC assay

The Annexin V/FITC assay was performed using Annexin V Kit (BD Pharmigen, USA) in order to analyse the potential of B1 AMCE in causing apoptosis. The cells were seeded in a 6-well plate at a concentration of 2.4 × 10^5^ cells/mL and incubated overnight. On the next day, the seeded cells were treated with the IC_50_ value of *Annona muricata* crude extract (AMCE) and incubated for 48 and 72 h. The cells were harvested according to the incubation time point and the resulting pellets were resuspended in the binding buffer provided. Five microliter of FITC Annexin V and 5 uL of PI were added to stain the cells suspension and allowed to stand in a dark place at room temperature for 15 min. Afterwards, the stained cells were analysed by flow cytometry machine (Becton Dickinson, USA).

### Acridine Orange/ Propidium Iodide assay (AO/PI)

Cell viability/apoptosis of the 4 T1 cells was analysed based on the AO/PI dual staining of live/dead nucleated cells. The AO/PI assay was carried out according to the protocol described by Salim et al. [10] with a slight modification. Cells were seeded in a 6 well-plate at a concentration of 2.4 × 10^5^ cells/mL and incubated overnight before treating with the IC_50_ value of *Annona muricata* crude extract (AMCE) the following day. The cells were incubated for another 72 h. Afterwards, the cells were harvested and the resulted pellets were resuspended in 200 μL PBS. Six microliter of the suspended cells was then stained with 4 μL AO/PI and the mixture were loaded onto a glass slide. The images were captured with a fluorescence microscope equipped with Nikon camera.

### Cell cycle assay

To further examine the effects of B1 AMCE on the induction of apoptosis, the effects on the cell cycle was tested. The cell cycle assay was carried out using CycleTEST PLUS DNA Reagent Kit (BD Pharmigen, USA). The cells were seeded at a concentration of 2.4 × 10^5^ cells/mL in a 6 well-plate and incubated overnight. The next day, the seeded cells were treated with IC_50_ of *Annona muricata* crude extract (AMCE) and incubated for 72 h. After trypsinization, cells were collected and a volume of 250 μL of solution A (trypsin buffer) was added. After 10 min of incubation at room temperature, 200 μL of solution B (trypsin inhibitor and RNase buffer) were added and the cell suspension was mixed gently. A further 10 min of incubation time at room temperature were required before a 200 μL of cold solution C (propidium iodide stain solution) were added to stain the cells. The mixture solutions were incubated for another 10 min in the dark on ice before analysed by flow cytometer machine (Becton Dickinson, USA).

### Migration/Invasion assay

This assay was attempted based on the predicament of the 4 T1 cells are able to migrate/invade with the presence of stimulants. It was conducted based on the protocol outlined by Chen [11]. Prior to the experiment, a 70 % confluent 4 T1 cells were serum starved for 24 h before being seeded at a density of 2 × 10^5^ cells/mL in the insert chamber coated with solidified Matrigel (BD Biosciences) for the invasion assay whereas for the migration assay, the chamber was not coated by the Matrigel basement membrane. In the lower compartment of the chamber, 2 mL of RPMI medium supplemented with 10 % FBS and the desired concentration of *Annona muricata* crude extract was added. The inserts were incubated in a 37 °C CO_2_ incubator for 24 h. The inserts were removed afterwards and the inner side of the inserts were swabbed to remove the non-/invaded cells. The outer side of the inserts bearing the migrated/invaded cells were then fixed in methanol for 30 min before being stained with 0.5 % of crystal violet. The images appeared on the membranes were later captured with an inverted microscope equipped with a camera (Nikon, Japan).

### Wound healing assay

This assay was done using the method outlined by Liang et al. [12]. A concentration of 3.5 × 10^5^ 4 T1 cells were seeded in a 6-well plate and incubated overnight. The next day, a straight wound line was drawn across the 100 % confluent attached cell layer with pipette tips. The floating cells were removed with PBS and replaced with new fresh RPMI medium. *Annona muricata* crude extract (AMCE) was added to the wells and images of the closure of the wound were recorded at time point 0, 3, 6, 9, 12, and 24 h using the inverted microscope equipped with a camera (Nikon, Japan).

### Animal and diet

Six to eight-week-old female BALB/c mice were used for in vivo experiments and were obtained from UPM Animal Resource Unit. Mice were divided into groups and acclimatized for 7 days, fed with normal diet and water. All methods involving the experimental use of animals have been reviewed and approved by the Institutional Animal Care and Utilize of Committee of the Faculty Veterinary and Medicine, Universiti Putra Malaysia (Reference Number: UPM/IACUC/AUP/RO55/2015). All animals were fully conducted in humane and ethical care and under the regulation of the governing body concerning the animals.

### Animal treatment

Mice were separated into 3 open-cages defining their respective groups; normal, untreated, and treated where each group bearing 7 mice per cage. Mice in the untreated and treated group were induced with 1 × 10^5^ cells/mL of 4 T1 breast cancer cells via subcutaneous (s.c) injection using a 27 gauge needle (Teruma, USA). Mice were observed on a daily basis for about 5 days until the tumor masses develop. Treatment with *Annona muricata* crude extract (AMCE) of 20 mg/20 g mice was given to the treated group while the other two groups were fed with distilled water. This treatment was conducted once daily for 28 days. After 28 days of treatment, the mice were euthanized and then sacrificed by cervical dislocation. Tissue samples like tumors and vital organs which include lung and spleen were harvested and directly used in downward analysis. One-half of the tumors were placed in tubes containing 10 % formalin for fixation and histological analysis while the other half were stored in tubes containing ‘RNAlater’ solution.

### Hematoxylin and eosin histology staining of the tumors

The harvested tumors were fixed in 10 % formalin and were embedded in paraffin before being sliced into thin sections. Then the paraffin sections were stained with hematoxylin and eosin (H&E) and were viewed under a bright-field microscope (Nikon, Japan). The mitotic cells present were counted and compared between the groups.

### Lung clonogenic assay

The metastasis of 4 T1 cells to other parts from primary tumor site was investigated by clonogenic assay. The clonogenic assay was carried out based on DuPre et al’s protocol [13]. Lung organ was harvested from the untreated and treated group of mice under sterile condition and was chopped into smaller pieces as to avoid the clogging of pipette. Afterwards, it was placed in the tubes containing 5 mL PBS and 100 uL (2 mg/mL) of collagenase type IV for 30 min at 37 °C. The solutions were passed through a 70 mm cell strainer and recollected in a new tube before centrifuging to obtain the pellet. The cells pellets were washed with PBS twice before being resuspended in 10 ml RPMI medium supplemented with 10 % fetal bovine serum and 60 μM 6-thioguanine (Fisher, USA). The cell suspension was plated in a 6-well plate and a 1/10 serial dilution was performed to fill the other 5 wells of the same plate. The plates were incubated for 10 days in a 37 ˚C incubator equipped with 5 % CO_2_. Unattached cells were rinsed with PBS twice before the attached cells/colonies were fixed in methanol for 1 h and later stained with 0.5 % crystal violet for another 1 h. The wells were washed by PBS and viewed under microscope.

### Immunophenotyping assay

The effect of B1 AMCE on the level of immune cells population from spleen was investigated by this assay. The spleens from the mice of all groups were harvested in a sterile condition. They were placed into a petri dish containing PBS solution and were mashed through 70 μL cell strainer. The single cell suspension were washed twice with ice-cold PBS and followed by the centrifugation step. The splenocytes were resuspended in a 2 mL NH_4_Cl lysis buffer and incubated for 10 min at 4 °C. Later, the cells were washed with PBS and centrifuged until a clean yellow pellet obtained. The pellets were dissolved in PBS and CD3, CD4, CD8, AND NK1.1 dye (Abcam, USA) were added into tubes accordingly in dark condition and were shook at 150 rpm for 2 h. Afterwards, 1 mL of PBS was added and the tubes were centrifuged. The resulted pellets were dissolved with 600 uL of 1 % paraformaldehyde and stored in the dark place at 4 °C before being analysed by flow cytometer machine (BD, USA).

### MDA antioxidant assay

The effect of B1 AMCE as antioxidant against lipid peroxidation in 4 T1 tumor sample was investigated on the basis of the level of malondialdehyde (MDA). Two hundred microliter of tumor sample supernatant was mixed with 800 μL of PBS, 25 μL of butylated hydroxytoluene (BHT), and 500 μL TCA. The mixture was vortexed and incubated on ice for 2 h. After centrifuging at 2000 × g for 15 min, 1 mL of supernatant was taken out and transferred into tube containing 75 μL of 0.1 M EDTA and 250 μL of 0.05 M TBA. The tube was boiled in water bath for 15 min and then, left to cool at room temperature before read by spectrophotometer at 532 nm and 600 nm wavelengths. The result obtained was compared to MDA standard curve.

### Nitric oxide/Griess reagent assay

The effect of B1 AMCE on the level of nitric oxide in 4 T1 tumor was investigated using the Griess reagent assay. It was carried out using the Griess Reagent Kit for Nitrite Determination (Life Technologies, USA). Twenty microliter of Griess reagent containing equal volume of sulfanilic acid and N-1-napthylethylenediamine dihydrochloride was mixed with 150 μL of the nitrite-containing sample and 130 μL of deionized water in a microplate and incubated for 30 min at room temperature. Standard curve was also prepared by diluting the nitrite standard solution with deionized water to give a series of concentration between 1–100 μM. In place of the nitrite- containing sample, the standards were mixed with the Griess reagent and incubated in a similar manner. The absorbance of the sample and standards were read by spectrophotometer (Beckman Coultor, USA) at 548 nm wavelength before the nitrite concentrations corresponding to the standard plot could be evaluated.

### Proteomic assay

The effect of B1 AMCE on the protein level affecting the angiogenesis process in 4 T1 tumor was investigated using the Raybio Mouse Angiogenesis Kit (RayBiotech, Inc.). A volume of 100 μL of 1x Blocking Buffer is added into each well of the glass chip and incubated at room temperature for 30 min. The Blocking Buffer were decanted and aspirated before 100 μL of samples were added into the wells and incubated for 2 h at RT. Later, the samples were removed and the wells were washed with Wash Buffer I for 3 times at each 2 min interval. The glass chip assembly was submerged into a container containing Wash Buffer I and shook gently for 10 min and this step was repeated with the Wash Buffer II. The wash buffer was decanted before 70 μL of 1X Biotin-conjugated Anti-cytokines were added into each wells and incubated with gentle rocking for 2 h at RT. After washing with Wash Buffer I and followed by Wash Buffer II, a 70 μL of 1X Streptavidin-Fluor were added to each well and incubated in a dark room for another 2 h in a similar manner. The washing steps were followed after removing the streptavidin-fluor from the glass chip. Later, the glass chip was removed from its tube assembly and rinsed with deionized water. A dry glass chip was sent immediately to scanning with laser scanner (Innopsys‘InnoScan) at excitation frequency of 532 nm.

### Statistical analysis

All data were expressed as the means ± standard error of mean (S.E.M.). The analysis was performed with one-way analysis of variance (ANOVA) and the group means were compared by Duncan test. Values of *p* < 0.05 were considered as statistically significant.

## Results

### Anti-proliferative effect of *Annona muricata* crude extract (AMCE) on MCF-7, MDA-MB-231, and 4 T1 cells

**Fig. 1:**
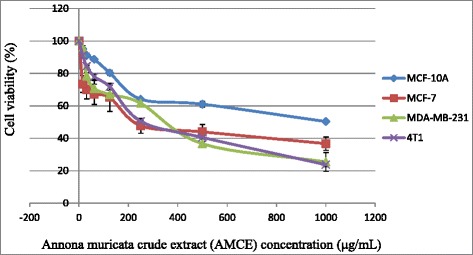
Representative MTT assay showing the cytotoxicity activity of B1 AMCE in three different types of cancer cells; MCF 7, MDA-MB-231, and 4 T1 and normal breast cell; MCF-10A after 72 h of incubation in vitro

Cell viability was determined by comparing to the survival of cells in the untreated (negative control) cultures, which was normalised to 100 %. The IC_50_ results for the anti-proliferative effect of the 19 samples of AMCE on three different breast cancer cell lines; MCF 7, MDA-MB231, and 4 T1 were shown in Table 1. The cells were treated with 2-fold serial dilutions of AMCE for 72 h. B1 sample was the most potent AMCE among others as it exhibited the lowest IC_50_ (half-maximal inhibitory concentration) for all breast cancer cell lines (MCF 7 = 220 μg/mL; MDA-MB231 = 350 μg/mL; 4 T1 = 250 μg/mL) as depicted in Fig. 1. Figure 1 also showed the IC_50_ of B1 AMCE on the positive control cell line, MCF-10A was considerably higher than the three cancer cell lines (1000 μg/mL). On the other hand, A2 and R2 samples were the least potent as both showed weak activity in inhibiting the proliferation of cancer cells as they have higher IC_50_ compared to the other ACME samples.

**Table 1:** The mean values of IC_50_ of all AMCE samples from MTT assay in MCF 7, MDA-MB-231, and 4 T1 cells

Sample	Breast cancer cell line		
	MCF 7	MDA	4 T1
J1	302.33 ± 1.45^b^	347.67 ± 1.45^a^	321.67 ± 7.27^c^
M1	348.33 ± 1.67^b^	360 ± 5.77^a^	312.33 ± 1.45^e^
M2	547.33 ± 3.93^f^	519.67 ± 2.91^f^	501 ± 3.05^h^
M3	349.33 ± 2.96^d^	496.67 ± 3.33^e^	450.67 ± 8.29^e^
M4	500.67 ± 6.36^d^	600 ± 1.16^g^	450 ± 10.41^g^
N1	251.33 ± 0.67^d^	353.67 ± 5.93^a^	449.67 ± 1.45^b^
N2	648.33 ± 4.41^g^	601.5 ± 4.44^g^	535 ± 7.64^j^
N3	550 ± 8.66^h^	601.67 ± 0.88^g^	599.33 ± 7.88^h^
B1	221.67 ± 1.67^a^	350 ± 5.77^a^	251.67 ± 6.01^a^
B2	249.33 ± 4.7^d^	379 ± 3.06^b^	449.33 ± 7.88^b^
B3	330 ± 1.73^c^	348.33 ± 4.41^a^	400 ± 8.74^d^
A1	349.33 ± 7.22^e^	461.33 ± 9.14^d^	472.67 ± 5.36^e^
A2	701.67 ± 4.41^i^	731.67 ± 0.88^j^	646.33 ± 1.86^k^
A3	453.67 ± 1.86^d^	431.67 ± 16.91^c^	443 ± 3.51^f^
A4	302 ± 1.53^c^	360.67 ± 5.81^a^	400.67 ± 2.96^c^
R1	702.67 ± 1.45^f^	670.67 ± 0.67^i^	501 ± 6.66^k^
R2	799.67 ± 0.88^h^	769.33 ± 7.06^k^	605 ± 5^l^
R3	650 ± 5.77^f^	654.33 ± 6.36^hi^	500 ± 7.64^j^
R4	620 ± 2.89^d^	648 ± 1.73^h^	452.67 ± 6.23^i^

Each data was expressed as mean ± standard error of mean (S.E.M) of triplicate determinations. Mean values with different superscripts in the same column are significantly different *p* < 0.05

### B1 AMCE sample induced apoptosis in 4 T1 breast cancer cells

**Fig. 2:**
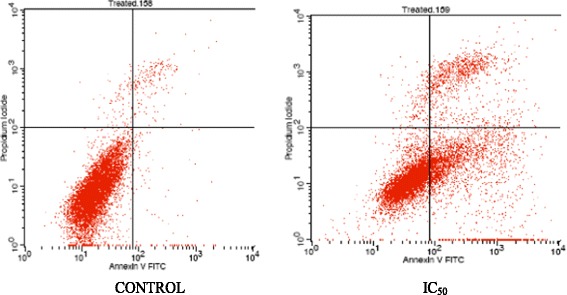
Histogram analysis of Annexin V/FITC in 4 T1 cells after being treated with IC_50_ concentration of *Annona muricata* crude extract (AMCE) after 48 h

**Fig. 3:**
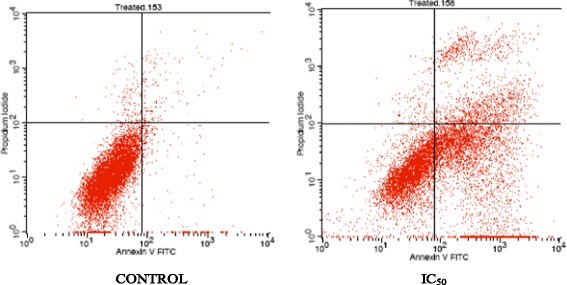
Histogram analysis of Annexin V/FITC in 4 T1 cells after being treated with IC_50_ concentration of *Annona muricata* crude extract (AMCE) after 72 h

**Fig. 4:**
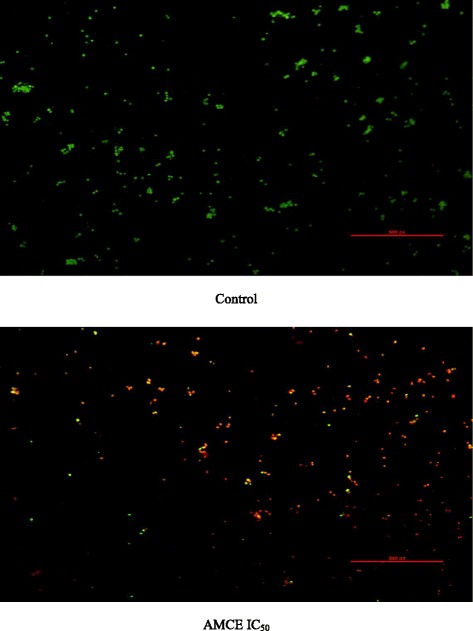
Images represent the control and treated cells which were stained with acridine orange and propidium iodide (AO/PI) after 72 h. 4 T1 cells were treated with IC_50_ of *Annona muricata* crude extract from B1 sample. Magnification: 100x

**Fig. 5:**
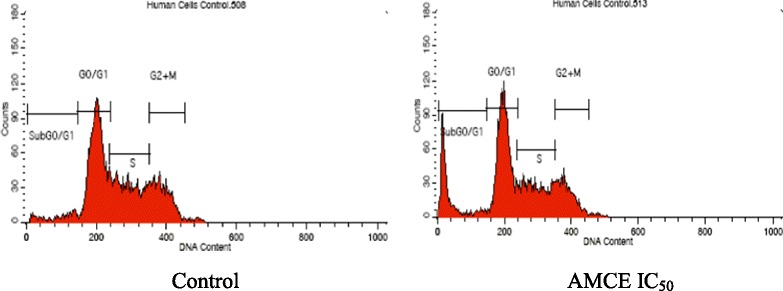
Histogram analysis of the cell cycle machinery in 4 T1 cells after being treated with B1 *Annona muricata* crude extract (AMCE) after 72 h

**Fig. 6:**
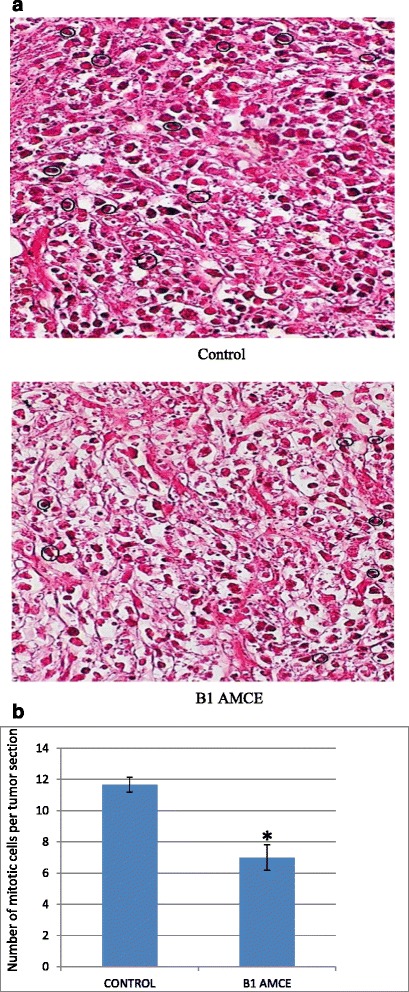
**a** Histological staining of both the tumors (control and B1 AMCE-treated) with hematoxylin and eosin (H&E). **b** Quantification of histological staining of the sectioned tumors of control and B1 AMCE-treated group. The dosage used in the treated group was 20 mg/20 g mice B1 *Annona muricata* crude extract (B1 AMCE). The data are expressed as means ± standard error of the mean for triplicates. Significance is set at *p < 0.05; *n* = 7 mice per group

Figures 2 and 3 showed the results of Annexin V FITC of 4 T1 cells after treating with the IC_50_ of B1 AMCE at two different time intervals; 48 h and 72 h respectively. The lower left quadrant of both histograms shows the population of the viable cells while the lower right quadrant represents the population of early apoptotic cells. The upper right quadrant represents the non-viable and late apoptotic/necrotic cells. There is a pattern of cell population shifting from viable cells to early apoptotic to late apoptosis/necrosis in both time-points. Based on Table 2, at the 48-hour time interval, the early apoptotic cell population increased gradually from 1.24 % ± 0.06 % in the control group to 26.9 % ± 1.18 % in IC_50_ of the treatment group whereas at 72-hour time interval, the cells increased from 2.39 % ± 0.09 % in the control group to 35.13 % ± 1.19 % in the treatment group. There was also an increase in population of late apoptotic cells from 1.89 % ± 0.03 % and 1.33 % ± 0.09 % in the control group to 10 % ± 0.08 % and 14.25 % ± 0.62 % in the B1 AMCE treatment group of the 48-h and 72-h time-point respectively. The difference of cell population percentage between 48 and 72-h time interval indicates that B1 AMCE was cytotoxic and induced apoptosis in time-dependant manner. These results (48 and 72 h treatment) coupled with the positive results shown in the in vivo tests (later will be discussed) that involved daily treatment for 28 days lead to the assumption that this crude extract is a potential candidate for treating breast cancer in human, as it can modulate its therapeutic effect continuously with no sign of resistance hence, effectively inhibit the growth of cancer cell in vitro and in vivo. Based on Fig. 4, the untreated 4 T1 cells were marked with distinct green intact nuclear structure colour which showed normal structure without prominent apoptosis and necrosis. On the other hand, the treated 4 T1 cells were consisted of cells population of bright green and orange-reddish colour as a result of the intercalation of AO and PI within the fragmented DNA, which represented the incidence of early apoptosis and late apoptosis respectively. To further study AMCE potential in inducing apoptosis, the cell cycle analysis was carried out using flow cytometric methods. Based on Fig. 5 and Table 3, after treatment with B1 AMCE (250 μg/mL) for 72 h, the percentage of cell population in the sub G0/G1 phase was significantly higher than in the control group (4.37 % ± 0.20 % versus 18.02 % ± 2.21 in the control group). This result suggested that B1 AMCE arrested the cell cycle at the sub G0/G1 phase and induced apoptosis in vitro. On the other hand, H&E images of the sectioned tumors as shown in Fig. 6 indicated a decrease in the number of mitotic cells (black circles) in the B1 AMCE-treated tumor compared to the control tumor. The low mitotic number means the decline of the number of actively dividing cells, thus, inhibit the cancer cells from sustaining themselves.

**Table 2:** Flow cytometric analysis of phosphatidylserine externalization on 4 T1 cells after 48 and 72 h of treatment

Treatment group		Percentage of cells (%)			
		Viable	Early apoptosis	Late apoptosis	Dead
48 h	Control	95.98 ± 0.15	1.24 ± 0.06	1.89 ± 0.03	0.89 ± 0.08
	B1 AMCE	61.48 ± 1.13*_a_	26.9 ± 1.18*_a_	10.01 ± 0.08*_a_	1.61 ± 0.1
72 h	Control	95.09 ± 0.24	2.39 ± 0.09	1.33 ± 0.09	1.18 ± 0.17
	B1 AMCE	49.39 ± 1.74**_b_	35.13 ± 1.19**_b_	14.25 ± 0.62**_b_	1.24 ± 0.14

Each data was expressed as mean ± standard error of mean (S.E.M) of triplicate determinations. *Statistical significance (*p*<0.05) between control and B1 AMCE-treated group in 48-hour time-point. **Statistical significance (*p*<0.05) between control and B1 AMCE-treated group in 72-hour time-point. Mean values with different subscripts in the same column are significantly different *p* < 0.05

**Table 3:** Percentage of cells in each of the cell cycle phases after treatment with B1 AMCE for 72 h

	Control	IC_50_(250_μg_/mL)
Sub G0/G1	4.37 ± 0.20^a^	18.02 ± 2.21^b^
G0/G1	45.23 ± 0.25^a^	43.12 ± 0.75^b^
S	29.98 ± 0.31^a^	23.48 ± 0.93^b^
G2 + M	20.55 ± 0.19^a^	15.39 ± 0.70^b^

Each data was expressed as mean ± standard error of mean (S.E.M) of triplicate determinations. Mean values with different superscripts in the same row are significantly different *p* < 0.05

### Anti-metastatic potential of B1 AMCE sample on 4 T1 cells

**Fig. 7:**
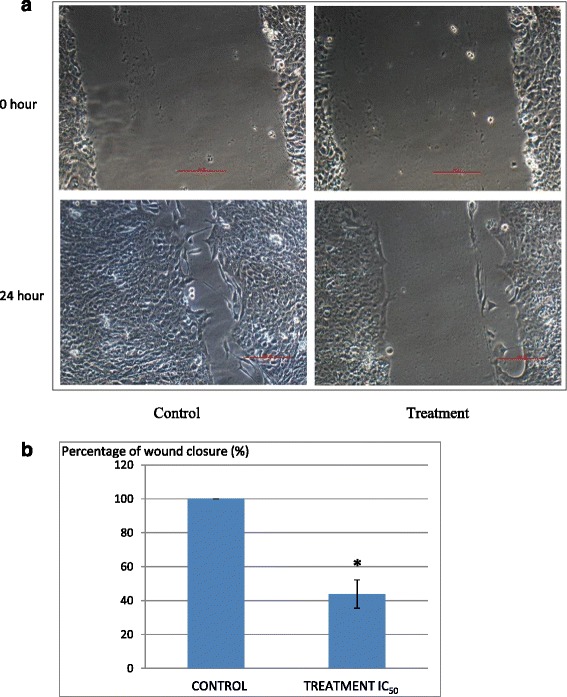
**a** Representative images of the wound healing analysis of 4 T1 cell at 0 h and 24 h for control and cells treated with IC_50_ value of B1 *Annona muricata* crude extract. Magnification: 100x. **b** Percentage of wound closure in 4 T1 cells when a wound is introduced. The assay was done in triplicates and the data are expressed as mean ± standard error of mean. Significance is set at *p < 0.05

**Fig. 8:**
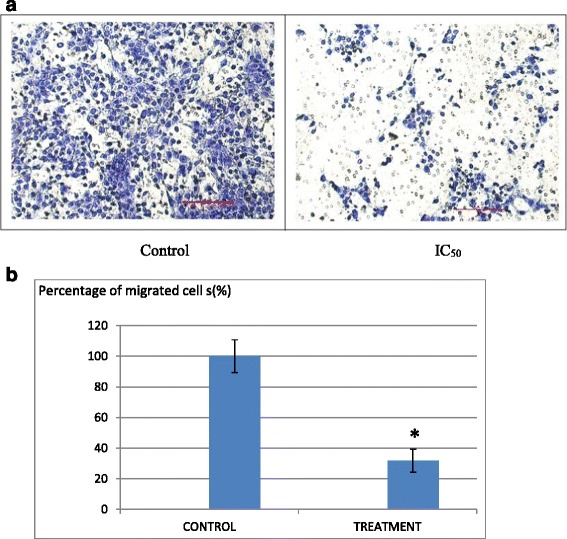
**a** Images of the in vitro migration analysis of 4 T1 cells; control and cells treated with IC_50_ value of B1 *Annona muricata* crude extract. The cells were allowed to migrate through an 8 mm pore membrane for 24 h. **b** Quantification of migration through 8-mm pore membrane inserts (BD Biosciences) by B1 AMCE-treated 4 T1 cells as a percentage of that achieved by control cells. The assay was done in triplicates and the data are expressed as mean ± standard error of mean. Significance is set at *p < 0.05

**Fig. 9:**
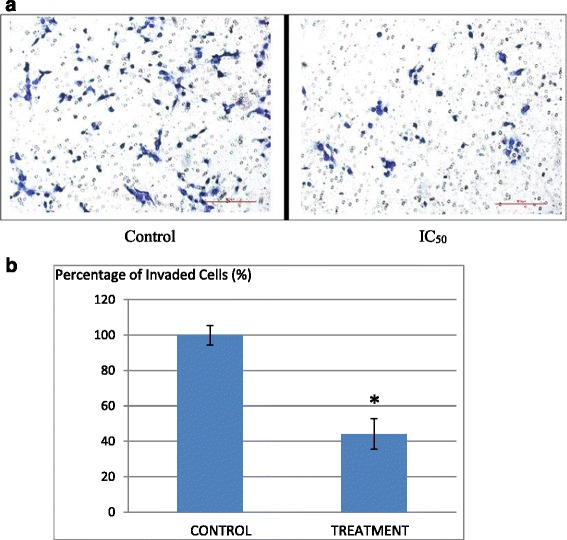
**a** Images of the in vitro invasion analysis of 4 T1 cells; control and cells treated with IC_50_ value of B1 *Annona muricata* crude extract. The cells were allowed to invade through a layer of Matrigel for 24 h. **b** Quantification of invasion achieved by the B1 AMCE-treated 4 T1 cells as a percentage of that achieved by control cells. The assay was done in triplicates and the data are expressed as mean ± standard error of mean. Significance is set at *p < 0.05

**Fig. 10:**
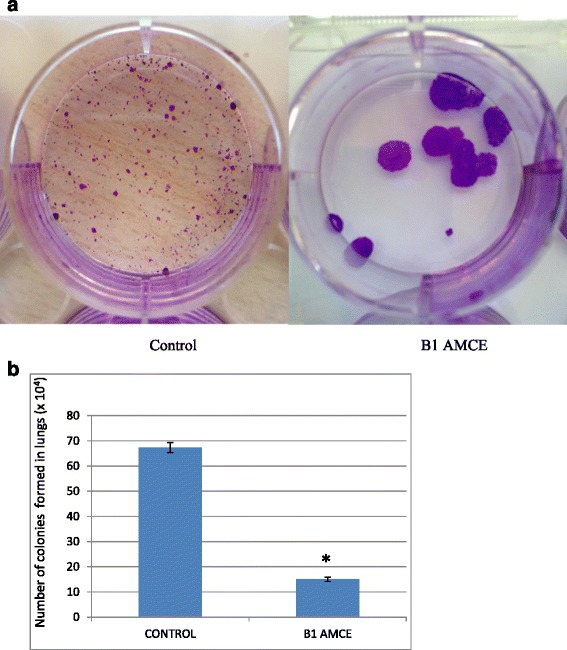
**a** Representative images of colonies formed in lung from clonogenic assay. Dilution factor: 10^-4^. **b** Total 4 T1 colonies formed from meshed lung harvested from the control and B1 AMCE (1 g/kg)-treated mice after 10 days of incubation. The data are expressed as means ± standard error of the mean for triplicates. Significance is set at *p < 0.05; *n* = 7 mice per group

**Fig. 11:**
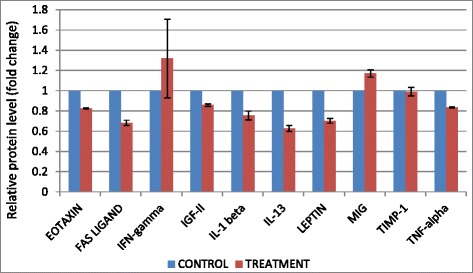
Significant changes of angiogenesis-related proteins level detected by proteome profiler when treated with B1 AMCE (1 g/kg)

In assessing the anti-metastatic abilities of B1 AMCE, in vivo clonogenic assay and in vitro assays like wound healing analysis, migration and invasion were carried out. In Fig. 7, it can be seen that there was a decrement in the percentage of wound closure in the B1 AMCE-treated 4 T1 cells, 43.9 % when compared to the control group, 100 %. In the migration assay, only 31 % of cancer cells managed to migrate through the transwell membrane when treated with B1 AMCE, as shown in Fig. 8b. This low percentage of migration rate in the treated cells compared to the control cells (100 %) indicated the potential of B1 AMCE to inhibit cancer cells migration. From the Matrigel invasion assay as shown in Fig. 9a, the ability of 4 T1 cells to invade a basement membrane was significantly compromised in the B1 AMCE-treated 4 T1 cells relative to the control cells. Quantifying this result, it was shown that treatment with B1 AMCE only allows 44 % cells to invade the basement membrane relative to the control cells (Fig. 9b). These results indicated that B1 AMCE significantly inhibited the migration and invasion of 4 T1 breast cancer cells in vitro. The metastasis of 4 T1 cancer cells in mice at the distant organ such as lung was determined via clonogenic assay as depicted in Fig. 10a. In Fig. 10b, the number of colonies formed in the lung was reduced significantly in the B1 AMCE treatment group (15 ± 0.82) compared to the control group (67 ± 2.05). In Fig. 11, several angiogenesis-related proteins were tested by proteome profiler in determining the proteins level of the B1 AMCE-treated group in relative to the control/untreated group (fold change). In comparison to the control group, the level of proteins such as Eotaxin, Fas Ligand, IGF-II, IL-1β, IL-13, Leptin, TNF-α, and TIMP-1 were decreased in B1 AMCE-treated group (0.83 ± 0.01, 0.68 ± 0.03, 0.86 ± 0.01, 0.76 ± 0.04, 0.63 ± 0.03, 0.70 ± 0.02, 0.83 ± 0.01, 0.990 ± 0.04) respectively. However, the level of proteins likes IFN-gamma and MIG were increased (1.32 ± 0.39 and 1.17 ± 0.03) respectively.

### B1 AMCE sample impeded the growth of tumor in vivo

**Fig. 12:**
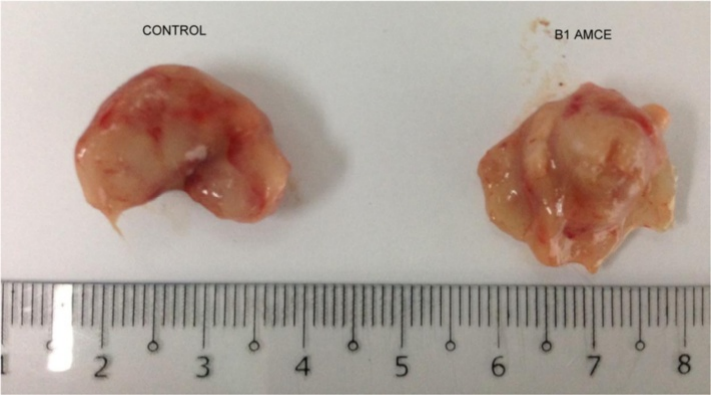
Images of tumors harvested from control and B1 AMCE (1 g/kg)-treated mice

**Fig. 13:**
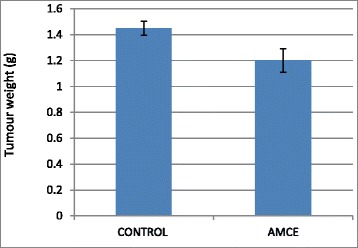
Weight of the tumors was measured after being harvested from the mice after 28 days of treatment. The data are expressed as means ± standard error of the mean for triplicates. *n* = 7 mice per group

**Fig. 14:**
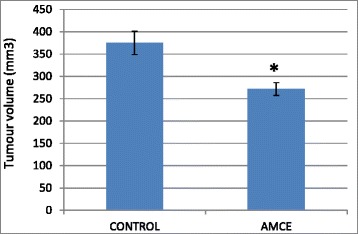
Volume of the tumors was measured using a vernier caliper. The data are expressed as means ± standard error of the mean for triplicates. Significance is set at *p < 0.05; *n* = 7 mice per group

The therapeutic effects of the B1 AMCE treatment in mice bearing the 4 T1-induced tumors were assessed after 28 days of treatment. Based on Fig. 12, the size of tumor in the control group are approximately similar to the B1 AMCE-treated group. However, the weight and volume of the tumor were different when compared between these two groups as shown in Figs. 13 and 14 respectively. In Fig. 13, the tumor weight decreased from 1.45 ± 0.06 g in the untreated group to 1.2 ± 0.09 g in the B1 AMCE-treated group. The mean tumor volume of the group treated with B1 AMCE was 271.7 ± 14.24 mm [3] which was smaller than the untreated group, 375 ± 25.98 mm [3] as depicted in Fig. 14.

### B1 AMCE sample regulated several immune systems markers in vivo and increase the level of white blood cell

**Fig. 15:**
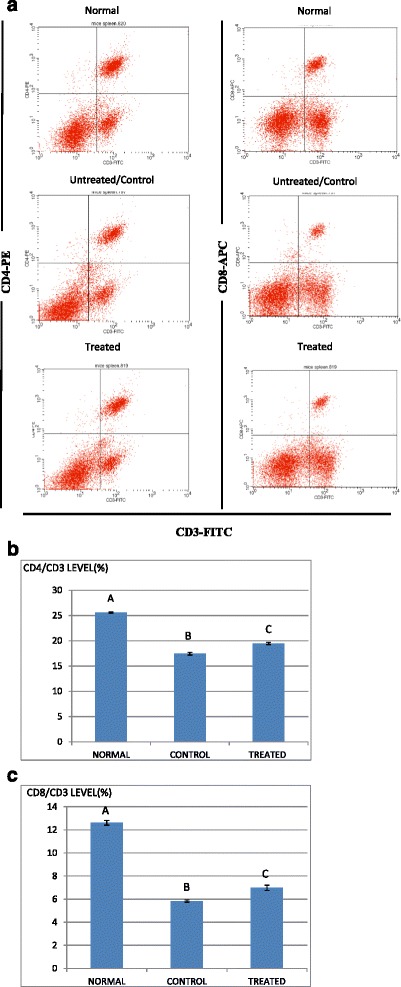
**a** Flow cytometry analysis of immune markers (CD3, CD4, and CD8) on the splenocytes of the normal, control, and B1 AMCE (1 g/kg)-treated mice. **b** Percentage of CD4/CD3 T cell population from the spleenocytes of the normal, control and B1 AMCE (1 g/ kg)-treated mice as depicted in Fig. 15**a**. The data are expressed as means ± standard error of the mean for triplicates. Mean values with different superscripts are significantly different *p* < 0.05; *n* = 7 mice per group. **c** Percentage of CD8/CD3 T cell population from the spleenocytes of the normal, control and B1 AMCE (1 g/ kg)-treated mice as depicted in Fig. 15**a**. The data are expressed as means ± standard error of the mean for triplicates. Mean values with different superscripts are significantly different *p* < 0.05; *n* = 7 mice per group

**Fig. 16:**
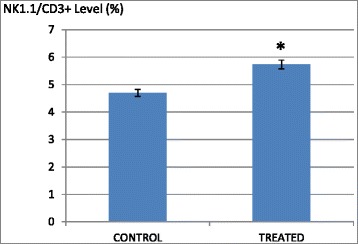
Percentage of NK1.1/CD3+ T-cell population from spleenocytes assay of the control and B1 AMCE (1 g/kg)-treated mice. The data are expressed as means ± standard error of the mean for triplicates. Significance is set at *p < 0.05; *n* = 7 mice per group

Immunophenotyping of the splenocyte cell population was carried out in order to gain the knowledge on the effect of B1 AMCE in modulating several important immune markers. In Fig. 15b, it can be seen that the splenocyte population of CD4/CD3-T cell was decreased from 25.6 % ± 0.11 % (normal group) to 17.46 % ± 0.28 % (control/untreated) but a significant increase was observed in the B1 AMCE-treated group (19.47 % ± 0.22 %). A similar trend was also observed in CD8/CD3-T cell population (Fig. 15c). A drop of CD8/CD3-T cell population percentage level was detected in the control group (5.83 % ± 0.10 %) when compared to the normal group (12.62 % ± 0.21 %) but the level of CD8/CD3- T cell population was elevated in the B1 AMCE-treated group (6.98 % ± 0.23 %). In addition, the population of natural killer (NK) 1.1/CD3^+^ cell was increased in the B1 AMCE-treated group (5.73 % ± 0.16 %) compared to the control/untreated group (4.69 % ± 0.13 %) as depicted in Fig. 16. Based on Table 4, the total white blood cell count observed was 4.5 × 10^9^ /L in the B1 AMCE-treated mice group which was higher than in the control group (2.4 × 10^9^ /L).

**Table 4:** Total white blood cell count in the serum harvested from the control and B1 AMCE (1 g/kg)-treated mice

MICE	Total white blood cell count, 10^9^ /L
Control	2.4
B1 AMCE	4.5

### B1 AMCE regulated inflammation

**Fig. 17:**
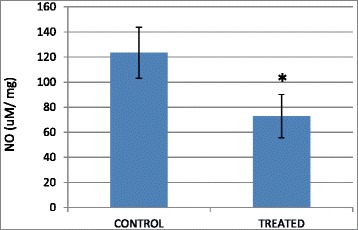
Level of nitric oxide in the tumors harvested from the control group and B1 AMCE (1 g/kg)-treated group. The data are expressed as means ± standard error of the mean for triplicates. Significance is set at *p < 0.05; *n* = 7 mice per group

**Fig. 18:**
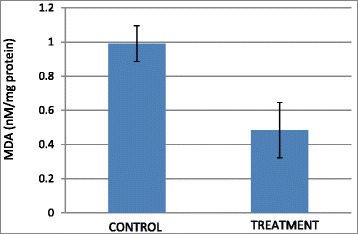
Level of malondialdehyde (MDA) in the tumors harvested from the control group and B1 AMCE (1 g/kg)-treated group. The data are expressed as means ± standard error of the mean for triplicates. *n* = 7 mice per group

The level of nitric oxide (NO) and malondialdehyde (MDA) in the tumor were assessed in both the control group and B1 AMCE-treated group. In Fig. 17, the level of nitric oxide marked a lower level of NO in the treated group (72.93 ± 17.12 μM/mg) compared to the control group (123.41 ± 20.29 μM/mg). A similar pattern was also observed in Fig. 18 where the MDA level was decreased from 0.99 ± 0.10 nM/mg in the control/untreated group to 0.48 ± 0.16 nM/mg in the B1 AMCE-treated group.

## Discussion

Natural products have been the target for cancer therapy for many years due to the medicinal values contained in them. In this study, the cytotoxicity effect of the aqueous leaf extract of *Annona muricata* Linn samples were evaluated on three different breast cancer cell lines; MCF-7, MDA-MB-231, and 4 T1 by MTT assay. Consistent with earlier findings [14], each of the soursop crude extract exhibited the anti-cancer activity as they inhibited the proliferation of the breast cancer cell lines as depicted in Table 1. The IC_50_ values are varied among the samples revealing the influence of the secondary metabolites constituents composed in them. This situation could be explained by the geographical difference of the sample cultivation area. The geographical difference of the cultivated plant means that each plant are exposed to different climate and environmental stress factors such as humidity, temperature, and soil composition [15]. The synthesis and accumulation of secondary plant products are enhanced in stress environment such as water deficit condition [16]. In harsh environment, plant adjusts their regulation of phenylpropanoid biosynthesis pathway at multiple levels in response to the exogenous factors. For example, green tea cultivated in area with high temperature, long sun exposure time, and high rainfall exhibits a higher concentration of theanine and lower concentration of leucine, isoleucine, epicatechin, and epigallocatechin compared to those cultivated in low temperature, short sun exposure time, and low rainfall [17]. Previous study had also shown that plants exposed in drought stress produce higher level of secondary metabolites indicating a crucial linkage between the environmental factor and metabolites contents [18]. In regards to those aspects, the cultivation area of B1 AMCE might be the harshest compared to the other samples hence, could be the underlying reason of its highest potency in killing cancer cells. Based on the cytotoxicity profile obtained, the aqueous leaf extract of soursop samples were more selective towards MCF-7 followed by 4 T1 and MDA-MB-231 cell line. As 4 T1 cell line was more aggressive than the other two cell lines and also to avoid any conflict of interest, 4 T1 cell line was chosen to be used in the downward assays. B1 AMCE sample which exhibited the best IC_50_ profile was selected for further use to treat the 4 T1 cells. Additionally, a successful anti-cancer drug should incapacitate cancer cells without causing excessive damage to normal cells thus, indicating minimal side effects. In this study, cell viability of normal breast cells, MCF10A in the presence of B1 AMCE treatment was determined. It appeared that B1 AMCE treatment was less toxic on normal cells as it required higher dosage to kill the cells (IC_50_ = 1000 μg/mL) which was four times higher than the IC_50_ of B1 AMCE in 4 T1 cells, thus, suggesting the low side effect of this plant crude extract. Flow cytometric analysis of Annexin V/FITC at 48-h and 72-h time-point distinguished a separate population of early apoptotic, late apoptotic/necrotic cells, and living cells as a result of the employment of the high affinity binding of Annexin V to phosphatidylserine (PS), a phospholipid component of the cell membrane. The dying cells which undergo apoptosis event experience a physiological change that causes the externalization of PS to the outer leaflet of the plasma membrane. As depicted in Table 2, the total apoptosis percentage (early apoptotic and late apoptotic/necrotic cells) in the B1 sample treatment group was higher than the untreated group. It is in accordance to the results presented in earlier report of apoptosis induction by soursop on colon cancer cells [19]. It is noteworthy that the B1 sample treatment induces the apoptosis in time-dependant manner where the apoptotic cells in 72-h time-point was found higher than in the 48-h time-point. As Annexin V/FITC analysis relies on the externalization of PS, the adoption of this AO/PI assay was purposely to detect different cellular event or morphological changes of the cells when treated with B1 AMCE sample. Apoptotic features such as membrane blebbing, nucleus condensation, and DNA fragmentation were evidently showed by AO/PI staining in the treated 4 T1 cells, thus strengthen the potential of soursop aqueous extract in inducing apoptosis and inhibiting breast cancer cells [20]. Subsequently, cell cycle analysis was also performed as the deregulation of cell cycle is closely related with apoptosis [21]. The regulation of cell cycle involves several checkpoint pathways to ensure the completion of one phase of the cell cycle before entering into another cycle phase in order to maintain the integrity of cell [22]. A significant increase of cell population at the sub G0/G1 phase was observed and shown in Table 3 which suggested the incident of cell cycle arrest in the B1 AMCE treated group. Halting the cell cycle progression in cancer cells eventually leads to the cell death which befits the idea of treating the breast cancer cells. Our data attested that B1 AMCE is capable of suppressing the tumor growth in the 4 T1 breast cancer in murine tumor models (after 28 days of treatment) based on the regression of weight and volume of the tumors, in agreement with the in vitro assays (MTT, Annexin V/FITC, AO/PI, cell cycle analysis) results. Additionally, according to the H&E staining of the tumors, the number of actively dividing cells (i.e., mitosis) which is a distinguished feature of cancer cells was reduced following the treatment of tumor with B1 AMCE when compared to the untreated control tumor. In order to consider that B1 AMCE as a potential candidate for antitumor drug, it is imperative that it possesses the capacity, by any mean, to inhibit the breast cancer cell from metastasize since the progression of tumor is not only dependent upon its proliferative rate. Metastasis which involves the migration and invasion of tumor cells has been long known as a formidable barrier to successful treatment. Therefore, in this present study, B1 AMCE had been put into test in vitro assays to justify this vital feature. In the wound healing assay, B1 AMCE managed to delay the growth of 4 T1 cells towards the centre of the wound which stressed out its propensity to prevent the migration of cancer cells. Hepatocyte growth factor/ scatter factor (HGF/SF), insulin-like growth factor II (IGF-II), and autotaxin which are among several factors reported to contribute to cancer cell motility [23] might be targeted by B1 AMCE but further clarification is required. This anti-metastatic effect was also well observed in the Transwell migration assay and the invasion assay where the number of cancerous cells was decreased in each assay in the presence of the B1 leaf extract. Evidently, this anti-metastatic potential of this plant extract is in accord with the previous finding although the setting of the study was on the pancreatic cancer cells [24]. Along with the in vitro studies, the in vivo studies were also carried out. The distribution of 4 T1 breast cancer cells to the secondary site such as the lung organ of the tumor-bearing mice was decreased in the B1 AMCE treated group as distinguished by the reduction of colonies formed in clonogenic assay. As can be seen in Fig. 10b, the colony formation was morphologically changed due to the B1 AMCE treatment. The formation of colony from ensembles of cells could be related to cell-cell adhesion and cell motility [25] thus, suggesting that 4 T1 cancer cells became less motile and more adhesive to each other in the presence of B1 AMCE treatment. However, it is noteworthy that there are no published data with specific attention have been reported to issue pertaining to the effect of the sample on colony size. Previous studies has identified that metastasis of tumor is made easier by the formation of new blood vessels at the surrounding matrix allowing a continuous interaction with other cells and systems of the body. As the multiple numbers of factors are involved in angiogenesis including the likes of growth factors, chemokines, cytokines, extracellular matrix macromolecule, and adhesion molecule, the present study was undertaken to observe the expression level of several angiogenesis-related protein in B1 AMCE-treated tumor harvested from tumor-bearing mice on the basis of the angiogenesis proteome profiler. The findings have shown that protein which includes the likes of Eotaxin, Fas ligand, IGF-II, IL-1β, TNF-α, IL-13, Leptin, and TIMP-1 were decreased when compared to the untreated tumor. Eotaxin, also referred as CCL11 is a chemokine that could foster angiogenesis through CCR3 receptor. It plays a critical role in inflammatory reactions; allergic and non-allergic as observed in previous studies and was also revealed to be a direct mediator of angiogenesis given the fact that it is an eosinophil chemoattractant which together with the eosinophilic products such as TGF-α and –β could induce angiogenesis [26]. Moreover, the level of Fas ligand was reduced significantly in the treated group compared to the untreated group. Fas ligand engagement with its receptor induces the apoptotic cell death and is important in modulating the homeostasis of cells in the immune system where its signal limits the expansion of T cell clones after the elimination of antigen [27]. In certain location of body such as the eye, testis, and placenta, Fas ligand is found highly expressed resulting in the death of invading Fas^+^ cells, including the lymphocytes which give them a privilege from immune surveillance [28]. Such strategy is also adopted by tumor cells to grant them an escape pass from being targeted by immune system thus, allowing them to successfully grow and proliferate. In addition, the level of insulin-like growth factor-II (IGF-II) is also dropped in the B1 AMCE-treated group. A mature IGF-II together with its homologous polypeptide sequence, IGF-1 and insulin, interact with either the type-1 IGF or insulin receptor located in the plasma membrane to transmit their growth promoting signals [29]. IGF-II level is found elevated in breast cancer patients and its involvement in cancer development could be seen through the MAPK pathway where IGF signal activates genes concerned with cell proliferation; and reduce the apoptosis event via the PI3-K/Akt pathway, hence, the occurrence of tumorigenesis [30]. Inflammatory cytokines such as tumor necrosis factor-α (TNF-α) and interleukin-1β (IL-1β) which are evidently contribute to angiogenesis, are also decreased in the B1 AMCE-treated group. In one study, these inflammatory cytokines alongside with inflammatory chemokines; CCL2 and CCL5 are expressed in a coordinated fashion which provides a combined role of the mediators to supports the growth and progression of breast tumor [31]. Another proangiogenic cytokine, interleukin-13 (IL-13) was also significantly less expressed in the treated group. IL-13 which is derived from T-lymphocyte is highly expressed in breast cancer as reported in previous studies and exerts its effect by inducing the up-regulation of VCAM-1 which consequently modulates the angiogenesis event [32]. Moreover, the level of tissue inhibitor of metalloproteinase-1 (TIMP-1), a member of the TIMPs family, was slightly decreased in the tumor treated group. The highly expressed TIMP-1 in breast cancer leads to tumor growth and development plus making the cells resistant to multiple apoptotic stimuli through the FAK/PI-3 K/AKT survival signalling pathways [33] despite its other role in inhibiting the MMP from degrading the extracellular matrix as demonstrated in other findings [34]. On the other hand, leptin which is frequently associated with obesity could also stimulate the proliferation of breast cancer cell lines as outlined in the previous studies [35, 36]. It is worth noting that its expression level was significantly reduced in the B1 AMCE-treated group. In spite of the decreased expression of several proteins, B1 AMCE could also up-regulates several proteins such as interferon-gamma (IFN-γ) and monokine induced by interferon-γ (Mig) which underlines its favourable criteria as anti-cancer agent. It has been discovered that IFN-γ has anti-tumoral effect as it manages to inhibit the growth of tumor cell lines including breast cancer cells by causing cell cycle arrest in the expense of p21 up-regulation as reported in previous studies [37] while in another findings, indicate that IFN-γ increases the growth inhibitory effect of tamoxifen in breast metastatic carcinomas [38]. The up-regulation of Mig in the treated group is a good indicator for B1 AMCE as anti-angiogenesis agent due to its ability to inhibit angiogenesis in vivo. In the presence of Mig, the neovascularization induced by the angiogenic factors such as IL-8, ENA-78, GCP-2, and GROα is inhibited [39]. Immune responses are responsible in the eradication of the neoplastic cells via the activation of the CD4^+^ and CD8^+^ T lymphocytes but a compromise to this barrier system could cost dearly. Based on the previous findings, tumor cells held its own machinery to evade from the immune surveillance by altering the activity of the T-cells thus, ensuring their progression [40]. From our study, it is apparent that the percentage level of the CD4^+^ and CD8^+^ T lymphocytes were dropped in the tumor group when compared to the normal group. This situation could be explained by the tumor-releasing Survivin as it has been described in one previous study. It was shown that Survivin, an apoptosis inhibitor, is released into extracellular space before eventually taken up by other surrounding malignant cells which describes their aggressive phenotype in terms of the increase of proliferative rate, resistance towards therapies, and their invasive potential. Survivin is taken up by T-cells as well due to its binding capability which consequently been the causal of the T-cells response polarization where proliferation and cytotoxicity of the T-cells are decreased [41]. Therefore, restoration of the T-cells level back to its normal state is necessary to combat and suppress the cancer cells. In our study, treatment with B1 AMCE in the tumor-bearing mice group marked an increase of CD4^+^ and CD8^+^ T lymphocytes population as well as the NK1.1 level compared to the control untreated group. Both CD8^+^ T-cell and NK cells are responsible in eliminating the cancer by lysing the tumors whereas T-helper cell is vital in further recruiting of both the aforementioned lymphocytes and also the cytokines for anti-tumor response purpose [42]. White blood cells are important in fighting infection and diseases which always appeared low in cancer patients [43] due to cancer itself that spreads beyond bone marrow site and displace the white blood cells or from the chemotherapy session. The increase in total white blood cell count may suggest the potential of B1 AMCE as a cancer therapy in recovering the white blood cell loss. Inflammation which is often related to immune modulatory response could initiate the progression of cancer once become chronic. One of the main culprits linking to this association is nitric oxide, a free radical product of NO synthase (NOS) where it is highly expressed in cancer cells and accounts for other multiple reactive intermediates [44]. Persistent expression of this mutagenic NO could contribute to tumor growth, metastasis, and angiogenesis as indicated in previous studies [45, 46]. Interestingly, B1 AMCE treatment exhibits a good therapeutic profile with a marked decrease of NO level within the tumor. Additionally, lipid peroxidation of polyunsaturated fatty acids is also induced in the wake of inflammatory response where it gives rise to several secondary products including malondialdehye (MDA), a highly toxic molecule [47, 48]. An intervention of the production of MDA is necessary to inhibit DNA damage and also to treat cancer, in overall perspective [49]. It is apparent that B1 AMCE could decrease the level of MDA within the tumor when compared to the untreated group thereby supporting the therapeutic potential of this leaf crude extract.

## Conclusion

Based on the results obtained from this study, it is imperative to carefully select the soursop samples from its cultivation area as it could determine the potency and anticancer activity of certain soursop sample. B1 AMCE has a good profile to be a candidate for breast cancer treatment as it managed to induce the apoptosis of 4 T1 breast cancer cells, inhibited the metastasis in vitro and in vivo, regulated the immune system, and reduced the inflammation caused by cancer. Nevertheless, a further evaluation of AMCE is needed to gain knowledge about its anticancer activity and mechanism.

## Additional file

Additional file 1: Table S1. Sampling sites of *Annona muricata* Linn in Peninsular Malaysia with the code and voucher number of each sample. (DOCX 14 kb)

## Abbreviations

AMCE: *Annona muricata* crude extract
AO/PI: Acridine orange/propidium iodide
MDA: Malondialdehyde
MTT: 3-[4, 5-dimethylthiazol-2-yl]-2,5 diphenyltetrazolium bromide
NH_4_Cl: Ammonium chloride
NO: Nitric oxide
PBS: Phosphate-buffered saline
