# Supplementary material for: Anti-cancer effect of Annona Muricata Linn Leaves Crude Extract (AMCE) on breast cancer cell line
Source: BMC Complement Altern Med. 2016 Aug 24;16(1):311. doi: 10.1186/s12906-016-1290-y (PMC4997662; doi:10.1186/s12906-016-1290-y)
Supplement: Supplementary file 1 [file 12906_2016_1290_MOESM1_ESM.docx]

Additional file 1

| LOCATION (STATE) | CODE (SAMPLING SITE) | VOUCHER NUMBER |
| --- | --- | --- |
| JOHOR | J1 (MUAR) | ATCL1 |
| MELAKA | M1 (JASIN) | ATCL2 |
| M2 (JASIN) | ATCL3 |
| M3 (BUKIT BERUANG) | ATCL4 |
| M4 (BUKIT KATIL) | ATCL5 |
| NEGERI SEMBILAN | N1 (KUALA KLAWANG) | ATCL6 |
| N2 (KUALA PILAH) | ATCL7 |
| N3 (KUALA PILAH) | ATCL8 |
| SELANGOR | B1 (SERDANG) | ATCL9 |
| B2 (SERDANG) | ATCL10 |
| B3 (SERDANG) | ATCL11 |
| PERAK | A1 (SUNGAI TERAP) | ATCL12 |
| A2 (KG. PIANDANG) | ATCL13 |
| A3 (KG. BATU TUJUH) | ATCL14 |
| A4 (KG BOTA) | ATCL15 |
| PERLIS | R1 (KAKI BUKIT) | ATCL16 |
| R2 (GUAR JENTIK) | ATCL17 |
| R3 (SANTAN) | ATCL18 |
| R4 (SANTAN) | ATCL19 |
